# Supplementary material for: Are carbon emissions trading and green financial instruments synergistic? -Comprehensive quantitative research based on content analysis
Source: PLoS One. 2024 Mar 7;19(3):e0298601. doi: 10.1371/journal.pone.0298601 (PMC10919601; doi:10.1371/journal.pone.0298601)
Supplement: S1 Data — (ZIP) [file pone.0298601.s001.zip › Data/地级市Z_F工作报告.zip!/╜¡╬≈/╕╙╓▌╩╨/└┤╫╘╣1⁄2╓┌║┼í╛┬φ┐╦╩2╛▌═°í┐_2018.docx]

各位代表：

　　现在，我代表市人民政府向大会报告工作，请予审议，并请各位政协委员和列席的同志提出意见。

　　一、2017年工作回顾

　　2017年是党的十九大胜利召开之年，是赣南苏区振兴发展史上极不平凡的一年。习近平总书记在全国深度贫困地区脱贫攻坚座谈会、《若干意见》【1】出台实施五周年和中宣部寻乌扶贫调研报告上，先后三次对赣州工作作出重要指示或批示，给予我们巨大鼓舞和鞭策。在党中央、国务院的深切关怀下，在省委、省政府和市委的坚强领导下，全市上下坚持解放思想、内外兼修、北上南下，突出打好六大攻坚战，纵深推进赣南苏区振兴发展，较好地完成了市五届人大二次会议确定的目标任务，取得了丰硕成果。

　　——获得了一系列来之不易的荣誉。全市人民众志成城，勇夺全国文明城市桂冠。荣获全国社会治安综合治理优秀市、综治“长安杯”。成功创建国家森林城市。赣南采茶歌舞剧《永远的歌谣》获“五个一工程奖”【2】。荣获2016年度全省科学发展综合考评第一名。全国就业扶贫、产业扶贫和全省工业、现代农业等31个现场经验交流会在赣州召开，在“中国制造2025”和全省脱贫攻坚、开放型经济、开发区创新发展等26个会议上作经验介绍，创赣州历史之最。

　　——推进和解决了一些重点难点事情。全市人民特别关注的昌赣客专赣州段隧道群全部贯通，赣深客专、兴泉铁路全线开工，广吉高速宁都段顺利推进，兴赣高速北延项目开工建设，黄金机场改扩建、航空口岸和通用机坪建设加速推进，华能瑞金电厂二期复工，省天然气管网赣州段6条支线开工建设，瑞金机场进场道路启动建设。市民期盼多年的和谐大道基本贯通，赣州火车站内部改造基本完工，文明大道等快速路加快建设，上犹江引水工程加快推进，市综合文化艺术中心开工建设，中心城区启动建设学校19所，新建数量超前五年总和。推行征拆新政，统一中心城区征拆补偿政策。妥善解决赣州经开区、蓉江新区征拆主体法律地位问题。拆除“两违”建筑500多万平方米，“两违”现象得到有效遏制。坚决依法推进农村“空心房”整治，累计拆除腾地2500万平方米。成功化解中祥·玖龙湾、义乌小商品城等信访难题。公立医院综合改革全面启动，持续半个多世纪的“以药补医”彻底终结。

　　——争取和落实了一些重大支持。充分发挥对口支援优势，争取上级各类补助资金530亿元以上，增长11%。获批国家山水林田湖草生态保护和修复试点，20亿元中央基础奖补资金全部到位。争取多年的东江流域生态补偿机制得到落实。获批全省唯一“中国制造2025”试点示范城市。信丰县入选国家首批现代农业产业园创建县。赣州港获批国家“一带一路”多式联运示范工程，成功开行中欧、中亚班列。省委、省政府出台纵深推进赣南等原中央苏区振兴发展、支持赣州建设省域副中心城市、打造“一带一路”重要节点城市等高含金量的政策，进一步提升了赣州的战略地位。

　　——出台和实施了一系列重大政策。推出促进经济平稳健康发展、培育经济发展新动能、支持大数据发展等一揽子政策措施，有效应对经济下行压力。推行“人才新政”【3】，启动5年建设10万套人才住房计划。颁布实施城市管理条例、农村住房建设管理办法。及时采取限购限贷限价等房地产调控措施。出台支持瑞兴于经济振兴试验区建设、“三南”园区一体化发展、蓉江新区加快发展、赣州港口岸物流发展等系列文件，区域发展活力进一步迸发。

　　一年来所做的主要工作及成效体现在：

　　一是六大攻坚战成效明显。实施六大攻坚战项目2056个，完成投资2504亿元，占年度计划的119%。270个市属重点工程全部开工，完成投资1415亿元，占年度计划的124.9%。预计全市GDP2500亿元，增长9.5%左右；固定资产投资2510亿元，增长13.8%；工业固投1043亿元，增长29%左右；服务业增加值1100亿元，增长12%；农村居民人均可支配收入9689元，增长11.0%，这5项指标增速有望保持全省第一。其他大部分指标增速保持全省前列。财政总收入408.32亿元，增长11.5%，一般公共预算收入245.36亿元，同口径增长8.6%。预计规上工业增加值增长9.2%；社会消费品零售总额890亿元，增长12.6%左右；实际利用外资16.67亿美元，增长10%；出口总额260亿元，增长16.3%；城镇居民人均可支配收入29470元，增长8.8%左右。

　　二是产业经济壮大升级。工业量质齐升。新增规上工业企业435户、总数达1721户，均列全省第一。高新技术企业达276家，占规上工业增加值比重33%。新开工标准厂房815万平方米，建成658万平方米。146个亿元以上“两城两谷一带”项目全部开工。“新能源汽车科技城”拉开框架、势头良好，国机智骏、山东凯马等7个整车及配套项目落户开工。“现代家居城”加速转型、提档升级，建成国内首个家具制造云——康居网【4】、全省首个家具设计中心，新增入规家具企业144家。“中国稀金谷”快速起步、创新发展，已入驻“国字号”创新平台4个，引进稀土和钨新材料及应用项目23个。“青峰药谷”建设加快、后劲增强，签约落地修正药业等16个重大项目，青峰药业获第二届井冈质量奖提名奖。“赣粤电子信息产业带”蓬勃发展、形势喜人，落户10亿元以上项目25个，引进合力泰等上市公司5家。各地首位产业聚力发展，上犹玻纤、于都服装服饰等10个省级重点工业产业集群加速壮大。农业结构加快调整。粮食生产“十四连丰”。蔬菜产业成为全省标杆、全国有影响，新建成规模蔬菜基地6.1万亩，开行中欧蔬菜班列。赣南脐橙名扬世界，列入中欧“100+100”互认保护【5】公示名单，稳居全国同类农产品区域品牌价值榜首。赣州成为全国油茶主产区。于都梾木油获批国家地理标志保护产品。服务业提速增效。金融机构存贷款余额分别增长17.9%、21.2%。利用贫困县IPO上市绿色通道引进拟上市企业34家，新增境外上市和新三板挂牌企业4家，趣店集团在美国上市。旅游接待人数、总收入分别增长22%、30.5%。方特主题公园、时光赣州、极地海洋世界等重大旅游项目加快推进。赣州综合物流园、赣州冷链物流中心等项目建设进展顺利，智慧物流“吉集号”上线运行。电商交易总额突破500亿元，增长60%以上。特色小镇建设全面展开、成效初显，全南南迳、宁都小布入选国家级特色小镇，大余丫山入围全国运动休闲特色小镇。

　　三是改革开放全面深化。“三去一降一补”【6】任务有效落实，提前完成省下达“十三五”钢铁去产能任务，超额完成煤炭去产能年度任务，降成本优环境行动为企业减负超100亿元。重点领域改革深入推进。“多证合一、一照一码”【7】登记制度改革实现24证合一。农村集体资产股份权能改革增点扩面，城乡户口“一元化”改登工作圆满完成。成立区块链票链全国监控运营管理中心，区块链金融产业沙盒园【8】启动建设，“瑞京金融”成为全省仅有的两家地方资产管理公司之一。经营性国有资产脱钩移交和统一监管工作基本完成，混合所有制改革和企业社区移交工作有序推进。创新平台加速壮大，国家离子型稀土资源高效开发利用工程技术研究中心、国家脐橙工程技术研究中心通过科技部验收，工信部电子五所（龙南）办事处、中科院海西研究院赣州稀金产业技术研发中心、中国稀金（赣州）新材料研究院挂牌运行，全省首个检验检测创新联盟组建完成，赣州工业设计中心、天翼·华为云计算数据中心建成运行。招才引智力度空前，成立赣南苏区人才发展合作研究院、市招才引智局，设立“苏区人才伯乐奖”【9】，组建同心圆智库，支持驻市高校融入地方发展。开放合作深入拓展。举办招商推介活动223场次，签约项目770个。引进绿地、TCL、招商局集团、杉杉集团等一批大企业、大集团，与深圳、广州、河源签署战略合作协议，与上海、广州、宁波、昆山等地开展国家级经开区结对共建。成立赣州商会联合总会，举办首届全国赣州商会联合大会。国际贸易“单一窗口”国家标准版试运行进展顺利。

　　四是城乡面貌大为改观。全市常住人口城镇化率达48.6%左右。中心城区五区融合发展，环境更加整洁有序。五洲大道、站东大道改扩建等工程竣工通车，东坡路等27条支路顺利打通，高铁新区、蓉江新区建设进展顺利。完成健康路、青年路、厚德路立面改造。115个老旧小区整治有序推进，1632条背街小巷提升改造全面启动。城乡环境整治强力推进。大力实施拆违治违、主次干道改造、公共服务设施完善等工程，大部分县城面貌焕然一新。农村生活垃圾基本得到有效治理。完成5797个新农村建设村点整治。实施国省道升级改造1144公里，建成农村公路2687公里，25户以上人口自然村全部通水泥（油）路，安远获评全国“四好农村路”【10】示范县。乡风文明行动扎实有效，农村面貌发生可喜变化。

　　五是生态屏障得到巩固。推进生态文明建设决心之大、力度之大前所未有。筹集183亿元实施国家山水林田湖草生态保护和修复试点，首批28个项目全部开工。完成废弃矿山治理19.1平方公里、低质低效林改造68.2万亩。东江流域出境断面水质达标率100%。完成中央环保督察反馈问题年度整改任务。“三净”【11】行动成效明显。空气质量保持优良。“河长制”【12】深入实施，8个省重点支持县（区）生活污水管网建设全面完成，重要水功能区水质达标率达到省级控制要求【13】，集中式饮用水源地水质达标率100%。在全省率先实施生态文明建设领导干部约谈制度，出台党政领导干部生态环境损害责任追究实施细则，安远、会昌、大余成立生态综合执法局，生态文明制度加快完善。

　　六是民生福祉持续增进。民生支出631.4亿元，增长14.3%，占一般公共预算支出的83.7%。脱贫攻坚成效显著。预计全年脱贫19.4万人、258个贫困村退出，瑞金脱贫摘帽扎实推进。赣南脐橙产业扶贫成为全国范例。就业扶贫车间基本实现乡镇全覆盖。完成易地搬迁5.4万人。健康扶贫“四道医疗保障线”【14】实现“先诊疗、后付费”和“一卡通”即时结算。贫困户子女助学补助基本实现全覆盖。省、市90件民生实事年度计划基本完成。新（改、扩）建公办幼儿园164所、义务教育学校439所、职业学校4所，已有12个县（市、区）通过义务教育均衡发展国家评估。棚户区改造基本建成2.4万套。完成农村危房改造2.76万户，其中农村保障房8710户。新（改、扩）建敬老院、光荣院、福利院60所。市人民医院新院全面投入使用，市妇保院新院开工建设。中心城区二级以上医院和县级医院实现跨省异地就医即时结算。城乡居民医保“一卡通”实现同步结算。机关事业单位养老保险制度改革稳步推进。安全生产、信访形势、食品药品安全状况稳中向好，公众安全感指数保持全省前列。国防、双拥、优抚、公安、消防、国土、审计、统计、人防、地震、民宗、外侨、对台、档案、地方志、妇女、儿童、老龄、残疾人事业等各方面工作取得新进步。

　　七是自身建设不断加强。深入学习宣传贯彻党的十九大精神，扎实推进“两学一做”学习教育常态化制度化，严格落实中央八项规定精神，深入推进政府系统改作风、提效率，行政效能明显提升。认真落实党风廉政建设主体责任，政府系统廉政建设不断加强。“放管服”改革持续深化。市本级行政审批事项精简至78项，为全省设区市最少。在全省率先推行“一窗式”【15】改革，正式运行市行政审批局。市县两级行政审批中介服务超市全面建成。法治政府建设不断加强，行政执法三项制度【16】国家试点顺利完成。自觉接受人大监督和政协民主监督，建议和提案办结率、满意和基本满意率均达100%。

　　各位代表，过去的一年，全市上下聚精会神谋发展、同心同德干事业、众志成城攻难关，取得了超乎预期的好成绩，在赣南苏区振兴发展的伟大征程中，书写了浓墨重彩的一笔！成绩来之不易，这是党中央、国务院亲切关怀和国家部委大力支持的结果，是省委、省政府和市委坚强领导的结果，是全市人民勠力同心、团结奋斗的结果。在此，我代表市人民政府，向辛勤奋战在全市各条战线的广大干部群众，向各位人大代表、政协委员，向各民主党派、工商联和无党派人士，向驻市人民解放军、武警官兵，向中央、省属驻市单位，向所有关心支持赣州发展的海内外朋友，表示崇高的敬意和衷心的感谢！

　　需向代表们说明的是，受全国投资增速放缓、去产能等因素影响，全市规上工业增加值、固定资产投资增速与预期目标还有少许差距。同时，我们还清醒地认识到，我市发展不平衡不充分的问题比较突出，经济总量不大，新经济发展较慢，创新能力不足；实体经济仍有不少困难，民营经济发展环境有待改善；脱贫攻坚任务艰巨，民生和公共服务领域还有不少短板；生态环保治理压力大；部分公务人员专业能力不强、作风不实、不敢担当。对此，我们将冲着问题改、迎着困难上，以昂扬向上的精神状态和一往无前的奋斗姿态，努力开创政府工作新局面!

　　二、2018年工作安排

　　2018年是贯彻党的十九大精神的开局之年，是决胜全面建成小康社会、实施“十三五”规划承上启下的关键一年，做好今年的工作意义重大。今年政府工作的总体要求是：全面贯彻党的十九大精神，以习近平新时代中国特色社会主义思想为指导，认真落实中央经济工作会议、省委十四届五次全会和市委五届四次全会精神，深入实施《若干意见》，按照高质量发展要求，坚持稳中求进工作总基调，坚持新发展理念，坚持以供给侧结构性改革为主线，深入贯彻省委“创新引领、绿色崛起、担当实干、兴赣富民”工作方针，坚持解放思想、内外兼修、北上南下，纵深推进六大攻坚战，统筹推进稳增长、促改革、调结构、惠民生、优生态、防风险各项工作，加快赣南苏区振兴发展，为决胜同步全面小康打下坚实基础，奋力打造新时代中国特色社会主义的红色样板。

　　2018年全市经济社会发展的主要预期目标是：GDP增长9%以上，规上工业增加值增长9.2%左右，财政总收入增长9%，一般公共预算收入增长6.5%以上，固定资产投资增长13%左右，社会消费品零售总额增长12.5%左右，实际利用省外资金增长15%以上，实际利用外资增长10%左右，出口总额增长4%左右，城镇、农村居民人均可支配收入分别增长8.8%左右和11%，居民消费价格总水平涨幅控制在3%左右，城镇登记失业率控制在4.5%以内，节能减排全面完成省下达计划任务。这些目标，既科学务实，又积极进取，体现了高质量发展要求，体现了“跳起来摘桃子”。我们要咬住目标不动摇，精心谋划，精准施策，精细落实，推动经济社会发展实现新跨越。重点抓好九个方面：

　　（一）坚决打好脱贫攻坚硬仗。在确保瑞金脱贫摘帽的基础上，力争全市脱贫21.5万人以上、贫困发生率降至2%以下、362个贫困村退出，南康、寻乌、安远、会昌、石城、上犹脱贫摘帽。

　　攻克深度贫困堡垒。倾斜支持167个深度贫困村和贫困群体，优先实施农村饮水安全、农村电网改造升级、宽带网络全覆盖等工程，倾斜安排危房改造指标，优先安排土地整治、增减挂钩等项目。落实深度贫困群体低保或五保全覆盖，民政救助、资金收益分红、公益性岗位向深度贫困群体倾斜。成立关爱深度贫困专项扶贫基金，深化“百企帮百村”，实现每个深度贫困村有1个企业结对帮扶。统筹推进城镇困难群众脱贫解困工作，确保全面小康路上一个都不能少。

　　提升扶贫工作实效。聚焦“收入达标、两不愁、三保障”，拓宽贫困户增收渠道，提高兜底保障水平。推进产业扶贫全覆盖，力争每个贫困村至少有1个扶贫产业基地或龙头企业。推进就业扶贫再扩面，增加公益性岗位，力争每个贫困村有扶贫车间。推进健康扶贫再提升，完善“四道医疗保障线”，探索重大疾病、慢性病、精神病、失能病人护理保险制度，实施贫困家庭医生签约服务全覆盖。推进搬迁扶贫再精准，完成易地搬迁3.5万人。推进教育扶贫再对接，确保适龄学生义务教育无因贫辍学。实施贫困村村庄整治再推进。坚持扶贫、扶志、扶勤、扶德、扶智并举，激发脱贫发展内生动力。

　　强化扶贫保障措施。完善脱贫攻坚责任体系，强化执纪监督问责，压实市县乡村和各行业部门脱贫攻坚责任。开展脱贫攻坚领域作风建设年活动，加强对驻村第一书记、工作队长和帮扶单位管理。深入推进财政涉农资金整合，完善扶贫项目资金拨付绿色通道，加强扶贫资金管理。建立返贫预警机制，有效防止系统性返贫。广泛动员社会力量扶贫济困，进一步构建社会扶贫大格局。

　　（二）决战主攻工业三年翻番。力争引进55个10亿元以上重大工业项目，实施亿元以上工业项目300个以上，工业固定资产投资突破1300亿元，坚决挺起工业脊梁。

　　加速形成产业集群效应。“新能源汽车科技城”要初具雏形，实现整车下线并投产，规划筹建汽车配套产业园，启动建设新能源汽车检测中心，积极创建智能网联汽车示范区。“现代家居城”要致力打造研发设计城、智能制造城、线上线下营销城和港口物流城，完成“南康家具”区域品牌申报注册，引进和培育一批国际国内品牌家具企业，力争主营业务收入突破1400亿元。“中国稀金谷”要围绕打造全国稀土和钨新材料研发生产基地，引进和培育1-2家稀有金属核心企业，做大做强中国南方稀土集团，力争全市稀土和钨产业集群主营业务收入突破1200亿元。“青峰药谷”要围绕打造大健康产业集群，推进产业链前延后伸，启动青峰药业二期、山香药业异地技改扩能等项目建设，引进5个亿元以上医药项目，新上市一批药品及医疗器械产品。“赣粤电子信息产业带”要围绕建设成为在全国具有较强竞争力的新兴电子产业基地，加快建链、延链、强链，引进电子行业巨头，力争主营业务收入突破500亿元。用好重大工业项目投资引导资金，引导各地聚焦发展首位产业，形成优势互补、错位发展新格局。

　　掀起新一轮园区创新发展热潮。创新营运模式，提升园区发展水平，实现工业园区在全国、全省排位前移。力争赣州经开区主营业务收入突破1000亿元，赣州高新区突破500亿元，龙南、瑞金经开区“一区多园”【17】分别突破600亿元、800亿元。实施“腾笼换鸟”工程，逐步腾退低效用地，清理“僵尸企业”【18】，促进节约集约发展。狠抓标准厂房建设，新开工建设500万平方米，累计建成1450万平方米。

　　做大做强骨干企业。壮大一批企业排头兵，力争培育40家以上主营业务收入过10亿元、2-3家50亿元以上的龙头企业，以及超百亿元企业。争取省级产业发展升级引导基金，整合市县两级工业发展资金，重点支持重大产业项目。大力推动“个转企、小升规、规改股、股上市”，新增规上工业企业300家以上，加快推进腾远钴业、金力永磁等企业上市。大力扶持“专精特新”企业【19】发展，培育一批细分市场占有率高的科技“小巨人”和“隐形冠军”企业。

　　（三）统筹推进区域协调发展。力争中心城区常住人口达180万左右，全市常住人口城镇化率达50%以上，加快建设省域副中心城市。

　　推进中心城区强心提质。加快中心城区五区一体化发展。建成文明大道、迎宾大道快速路，加快建设东江源大道等4条快速路，新开工赣南大道等3条快速路。推进城市主干路建设，今明两年完成73条主次干道和支路“白改黑或白加黑”【20】，建成红旗大道东延、东江源大道北延项目，实施和谐大道东西延伸、105国道中心城区段改线工程，打通营前路等7条支路。完善过江交通体系，拓宽改造南河大桥，新建蟠龙大桥等7座大桥，新建凤岗大道—蓉江四路过江隧道。高标准建设蓉江新区，推进赣州西站及周边市政基础设施建设，打造高铁商务区，加快建设市综合文化艺术中心，启动建设奥体中心、国际会展中心。实施城市修补和生态修复，启动古宋城核心区征地拆迁和“三江六岸”污染企业搬迁工作。加强城市立体综合开发，加大海绵城市、地下综合管廊建设力度。实施拆墙透绿增绿，推进立体绿化、屋顶绿化。大力发展装配式建筑。推进文明城市常态化精细化管理，推动县城创建文明城市，促进全市人居环境整体大提升。

　　壮大区域经济板块。加快瑞金、龙南次中心城市建设，争取完成龙南撤县设市。做大做强瑞兴于经济振兴试验区首位产业，推进瑞兴于快速交通走廊等项目建设，大胆开展先行先试，打造“老区中的特区”。深入推进“三南”园区一体化发展，加快“三南”快线等基础设施建设，推动共建“三南”承接加工贸易转移示范地产业园。探索会寻安生态经济区建设。谋划建设高铁经济带。高起点规划建设临港经济区。积极创建国家级、省级特色小镇，加快赣南脐橙、南康家居、上犹油画小镇等建设。

　　强化基础设施支撑。续建昌赣、赣深客专和兴泉铁路，抓紧瑞梅、长赣、赣郴永兴铁路和南丰至瑞金城际铁路、赣韶铁路扩能改造、赣广高铁等项目前期工作。加快广吉高速宁都段、兴赣高速北延项目建设，开工建设大广高速南康至龙南段扩容工程，做好信丰至广东南雄、寻乌南桥至广东龙川高速公路前期工作，争取井冈山至大余、寻全高速西延、赣州南至安远版石等项目纳入省高速公路网规划。完成黄金机场改扩建和通用机坪建设，加快建设航空口岸，全面推进瑞金机场建设。新开工国省道升级改造项目422公里，新改建农村公路2500公里。加速华能瑞金电厂二期扩建，开工建设神华信丰电厂，续建赣州西500千伏等输变电工程，启动新一轮农村电网改造升级。快速推进省天然气管网赣州段工程建设，所有支线年内全部开工，6条在建支线建成通气。加快推进城镇防洪工程建设，新建67个中小河流治理和8个五河治理工程。续建上犹江引水工程，新建定南洋前坝、兴国洋池口水库，利用三年时间实现县县有应急备用水源。

　　（四）大力实施乡村振兴战略。科学制定乡村振兴战略规划，建设产业兴旺、生态宜居、乡风文明、治理有效、生活富裕的美丽乡村。

　　做强做优特色产业。推进蔬菜产业迅速壮大，力争新（扩）建经济适用的生产性大棚10万亩左右，每个乡镇新建大棚200亩以上。推进脐橙产业发展升级，稳定种植面积150万亩左右，柑橘黄龙病平均病株率控制在5%以下。推进油茶产业改造提升，改造低产油茶林和新造高产油茶林各10万亩。巩固赣南脐橙品牌，打响赣南蔬菜、赣南茶油、赣南茶叶区域品牌。加快黄鸡、灰鹅、麻鸭、白莲、刺葡萄等县域特色产业发展。因地制宜发展林菌、林药、林养等林下经济，打造绿色兴农新亮点。

　　提升现代农业水平。严守耕地红线，完成33万亩以上高标准农田建设。强化龙头带动，把信丰国家级现代农业产业园、赣县国家现代农业示范区建成一流园区。引导农村土地经营权规范有序流转，推动解决耕地撂荒问题，发展多种形式适度规模经营，培育新型农业经营主体。大力发展农产品精深加工，力争新增规上农产品加工企业25家以上、亿元以上龙头企业5家以上。深入实施绿色农业十大行动，推进休闲农业和乡村旅游示范创建，加快发展智慧农业，培育农业农村发展新动能。

　　推进美丽乡村建设。扎实开展“整洁美丽、和谐宜居”新农村建设，抓好5000个左右村点整治。积极开展田园综合体建设试点，打造一批赣南特色田园乡村。实施农村人居环境整治三年行动计划，大力整治圩镇环境，加强农村污水、生活垃圾治理。完成农村“空心房”整治扫尾工作，对危旧“空心房”做到应拆尽拆、全面整理利用。加强农民住房建设管理，保护和修复传统村落和历史文化名村。提升乡风文明行动实效，深入推进移风易俗。做好第十届村（居）民委员会选举工作，加强乡村治理体系建设，提高村民自治水平。

　　（五）着力引爆现代服务业。促进服务业总量扩大、提档升级，建立与区域性中心城市相适应的现代服务业体系。

　　大力发展全域旅游。深入实施全域旅游三年行动计划，力争全年旅游接待人次突破1亿、旅游总收入突破1000亿元。按照“一核三区”【21】布局，推进江南宋城全域旅游景区、红色文化创意产业园等116个重点旅游项目建设。加快完善要素配套，加大旅行社扶持力度，每个县（市）新建1家四星级以上酒店，中心城区建设3家以上五星级酒店。实施景区提升工程，积极创建石城、龙南、瑞金国家全域旅游示范区，推动安远三百山、石城通天寨、大余丫山创5A级景区。深入推进“旅游+”，促进旅游与健康养老、文化创意、城乡建设、信息技术等领域深度融合。大力发展智慧旅游，实现旅游服务智能化全覆盖。

　　繁荣活跃金融商贸。加大实体经济信贷投放力度，力争新增银行贷款660亿元以上。推进赣州银行引进战略投资者，完成瑞京金融资产管理公司、金盛源担保集团组建运行。启动建设蓉江新区金融商务区与总部经济区。大力发展绿色金融【22】，推动发行绿色债券，重组赣州环境能源交易所。强化金融监管，坚决守住不发生区域性金融风险的底线。建设杉杉奥特莱斯、高铁站前商务中心等城市综合体。建成运营赣州综合物流园、赣州冷链物流中心一期工程，抓好赣州物流大数据产业园落地启动，力争打造国家物流交易结算中心。加快发展电子商务，实现电商交易额增长20%以上。

　　培育壮大特色康养产业。实施健康养老产业发展三年行动计划，推进寻乌青龙岩养生旅游等10大引领项目、玉虹文化苑养老服务基地等154个重点项目建设，引进一批高端医疗、健康管理和中医养生项目。深入开展医养结合试点，启动实施全国居家和社区养老服务改革试点，支持符合条件的医养结合型养老机构纳入医保定点范围，打响“善孝苏区·康养赣南”医养结合品牌。

　　（六）加快建设创新赣州。积极创建国家创新型城市、“中国制造2025”国家级示范区，打造江西科技创新次中心城市。

　　推动业态创新。大力发展智能制造，成立智能制造联盟，引进一批智能装备研发生产和集成服务企业。加快发展绿色制造，培育1个国家级绿色园区、2个国家级绿色工厂。积极发展服务型制造，建设赣州经开区新能源汽车、于都纺织服装等特色工业设计中心。启动建设蓉江新区大数据产业园、新一代信息技术产业研究院、章贡区软件和物联网产业园，引进一批大数据开发运用企业。组建产业与科技创新研究院，促进科技成果转化和产业转型升级。

　　夯实创新平台。深入实施创新驱动“1122”工程【23】，力争新建省级以上创新平台8个、新增国家级高新技术企业50家、R&D【24】经费支出占GDP比重不低于1.2%。加强产学研合作，创建钨资源高效开发与利用国家重点实验室。支持江西理工大学新校区及新能源汽车研发中心建设、组建新能源汽车学院，支持赣南医学院更名赣南医科大学、申报国家级油茶工程中心。争取青峰药业获批国家企业技术中心、金信诺等企业获批省级工程研究中心。积极争创国家军民融合创新示范区，建设省级军民融合有色金属新材料产业基地。深入开展质量提升行动，加快建设全国质量强市示范城市。

　　优化“双创”环境。加大招才引智力度，全面落实“人才新政”，开工建设人才住房2万套。推进国家知识产权试点城市建设，实现专利申请量增长25%以上。实施大学生创业引领计划，探索建立环大学创新创业带【25】。实施民营经济腾飞计划，建立民营经济投资环境测评体系，积极营造有利于企业家健康成长的良好环境。

　　（七）坚定不移深化改革开放。以纪念改革开放40周年为契机，迈出改革开放新步伐，构建更具活力、更加开放的发展环境。

　　深化重点领域改革。纵深推进“放管服”改革，精准实施相对集中行政许可权改革试点，推进“互联网+政务服务”和“网上办公”，提升“最多一次办结”改革事项比重，建设“多规合一”【26】信息管理平台。深化农业农村改革，全面完成房地一体的农村宅基地和集体建设用地使用权确权登记发证工作，有序推进农村集体经营性资产股份合作制改革，推动“三权分置”【27】政策落实。深化国资国企改革，推动混合所有制经济发展，完成市属国有企业改革重组，做大做强若干市属国有企业集团。深化投融资体制改革，大力促进社会投资，强化政府债务管理，加快存量债务化解，严格控制新增债务，推进融资平台市场化转型。深化医药卫生体制改革，实施公立医院人事薪酬制度改革，加快建立现代医院管理制度，推进市级医联体【28】建设，每个县（市）建立1个医联（共）体，建立分级诊疗【29】新模式。深化财税金融体制改革，全面实施绩效管理，开展县域金融工程试点，争创普惠金融改革试验区。稳妥推进从事生产经营活动事业单位改革。完成陡水湖、通天岩、赣江源等景区管理机制改革。研究完善市区两级财税、城建等管理体制，推进城管、文化等领域综合执法体制改革。推进社会信用体系建设，建成运营市级公共信用信息平台。加快建立多主体供应、多渠道保障、租购并举的住房制度，增加住房有效供应，稳定住房价格，促进房地产市场持续平稳健康发展。

　　精准高效招商引资。围绕首位产业补链强链，紧盯重点区域，锁定目标企业，重点对接行业前30强企业，着力招大引强。充分发挥赣州商会联合总会作用，扎实推进赣商赣才回归。实施招商引资项目落地行动，力争全年实际利用省外资金856亿元以上、实际利用外资18.3亿美元以上。

　　打造内陆双向开放高地。积极对接粤港澳大湾区、海西经济区，推动赣粤、赣闽产业合作区建设取得实质性突破。加快赣州港建设，建成运营赣州国际港站二期项目，申报进境肉类、汽车整车进口等指定口岸，常态化开行铁海联运快速货运班列和中欧、中亚班列。力争建成瑞金、龙南陆路口岸作业区。加快推动全市口岸一体化，逐步形成以赣州港为龙头的“1+N”大型内陆国际港，把赣州打造成为“一带一路”重要节点城市和国际货物集散地。

　　（八）持续加强生态文明建设。坚决打好污染防治攻坚战，筑牢南方地区重要生态屏障，争当生态文明试验区建设排头兵。

　　强力推进生态保护修复。统筹推进山水林田湖草生态保护和修复试点。深入推进东江流域生态补偿试点，确保出境断面水质稳定达标。强化森林资源保护，加强水土保持，推进森林质量精准提升示范市建设，完成低质低效林改造110万亩。坚决完成中央环保督察反馈问题年度整改任务，力争年内基本完成历史遗留废弃稀土矿山治理。加快绿色矿业发展示范区建设，深入开展园区循环化改造试点。纵深推进城乡环境整治，大力开展“厕所革命”，积极探索实行垃圾分类，推动城乡环卫一体化。

　　坚决向污染宣战。严格落实“大气十条”【30】，加大“四尘三烟三气”【31】巡查整治力度，在全市开展城市道路和建筑工地扬尘污染治理，全面禁止露天焚烧，打赢蓝天保卫战。全面落实“水十条”【32】，推进城市建成区水体质量提升工程，完成200公里城区生活污水管网建设改造，全面完成工业园区污水处理设施建设，全面关停拆除禁养区内规模化养殖场，坚决消灭劣Ⅴ类水【33】。深入实施“土十条”【34】，推进土壤污染管控修复计划，建成运行市生活垃圾焚烧发电厂、污泥无害化处置等项目，加快推进工业固体废物处置中心、重金属污染重点防控区项目建设。加强农业面源污染治理，提升土壤环境承载力。

　　实行最严生态保护制度。深入开展生态文明体制改革综合试验，推进自然资源资产管理体制改革。改革生态环境监管体制，完成环保机构监测监察执法垂直管理改革，推动组建县级生态综合执法机构。完善生态文明考核评价制度，严格执行生态文明建设领导干部约谈制度，深化领导干部自然资源资产离任审计试点，坚决用最严的制度守护绿水青山，实现永续发展。

　　（九）提高保障和改善民生水平。把群众的难事当作政府的大事，着力解决出行难、上学难、就医难等民生痛点，不断增强人民群众的获得感、幸福感、安全感。

　　扎实办好民生实事。在完成省下达民生实事任务基础上，筹资400亿元办好市40件民生实事。实施中心城区公共服务设施提升三年行动计划，打造10分钟便民生活圈。完成中心城区背街小巷提升改造。实施74条主次干道夜景亮化工程。新建停车场12个，年内建成6个。全面完成省“公交城市”创建目标。新建一批市民休闲公园、社区蔬菜便利店、农贸市场。实施城乡学校建设三年行动计划。新（改、扩）建幼儿园100所、小学42所、初中23所，完成297所薄弱学校改造。中心城区每个区新（扩）建1所普通高中，全市新（扩）建普通高中12所。建成赣南职业技术学院，新（扩）建中职学校5所。启动全国卫生城市创建。实施提升卫生计生服务能力三年行动计划。全面启用赣南医学院一附院黄金院区，加快市妇保院新院、市儿童医院、市儿童福利院、市精神卫生社会福利院建设，改造市人民医院老院区，建设市中医院新院。推进社区、乡镇、村医疗卫生服务机构标准化建设，实现每个行政村至少有1所标准化村卫生计生服务室。

　　大力发展社会事业。深入推进县（市、区）公共文化服务标准化、均等化三年建设计划，加快实施赣南围屋三年抢修计划，深入挖掘赣州红色文化、客家文化、阳明文化、禅宗文化，提升文化软实力。培育体育品牌赛事，创建全国运动休闲城市，申办2020年全国阳光体育大会。稳妥推进机关事业单位养老保险制度改革，全面实施全民参保计划，完善城乡居民基本医疗保险和大病保险制度。实施新一轮棚改三年行动计划，力争三年任务两年完成。加大社会救助力度，大力发展慈善事业，实施残疾人关爱工程，让每一位身处困境者都能沐浴社会关爱的阳光！

　　全力维护社会稳定。加强和创新社会治理，构建共建共治共享新格局。深入推进法治赣州建设，深化司法体制综合配套改革。提高平安赣州建设水平，推进社会心理服务体系和“雪亮工程”【35】建设，健全社会治安立体化防控体系。开展重点项目建设环境专项整治，严厉打击围标串标、阻工堵路、强揽工程等违法犯罪行为。深化信访制度改革，构建矛盾纠纷多元化解体系。严格落实安全生产责任制，坚决遏制重特大安全事故发生。深入创建国家食品安全示范城市，保障人民群众饮食用药安全。认真做好民族、宗教工作，促进民族团结进步、宗教领域和谐稳定。

　　各位代表，民之所望，政之所向。我们将切实加强政府自身建设，努力建设人民满意政府。我们将旗帜鲜明讲政治，牢固树立“四个意识”，坚决维护党中央权威和集中统一领导，坚决贯彻省委、省政府和市委各项决策部署，认真开展“不忘初心、牢记使命”主题教育。我们将依法行政重法治，加快转变政府职能，加强法治政府建设，推进重点领域政府立法，坚持依法科学民主决策，严格规范公正文明执法，自觉接受人大、政协、监察、司法、社会、舆论等各方面监督，让权力在阳光下运行。我们将驰而不息改作风，严格执行中央八项规定实施细则，坚定不移反对“四风”，大兴调查研究之风。深入落实市委、市政府“一个意见、两个办法”【36】，组织开展“大众评公务”活动，以敢担当、提水平为主题，纵深推进改作风、提效率。我们将弘扬专业精神抓落实，大力建设学习型政府，提升专业素养，增强工作本领，建设高素质专业化队伍，提高理解力、操作力、执行力，争创新时代“第一等工作”。我们将廉洁从政守底线，坚决落实全面从严治党要求，切实履行党风廉政建设主体责任，坚决查处损害群众利益的“害群之马”，坚持做到干部清正、政府清廉、政治清明。（下转3版）　　（上接2版）每一位政府工作人员都要以钉钉子的韧劲、言出必行的干劲，办好每件事，干好每一天，走好每一步，决不辜负党和人民的信任与重托！

　　各位代表，新征程赋予新使命，新时代呼唤新作为。让我们更加紧密地团结在以习近平同志为核心的党中央周围，坚持以习近平新时代中国特色社会主义思想为指导，在省委、省政府和市委的坚强领导下，不忘初心，牢记使命，为决胜全面建成小康社会、奋力打造新时代中国特色社会主义的红色样板而努力奋斗！
